# Impact of VP2 mutations on viral fitness in canine parvovirus

**DOI:** 10.1007/s42770-026-02035-2

**Published:** 2026-07-27

**Authors:** Tamiris Silva Lopes, Brenda Picoli Gheno, Ahmed Abd El Wahed, Uwe Truyen, André Felipe Streck

**Affiliations:** 1 Diagnostic Laboratory of Veterinary Medicine, Biotechnology Institute, Universidade de Caxias do Sul, Rua Francisco Getúlio Vargas, 1130, Campus Sede, RS Caxias do Sul, Brazil; 2 Institute for Animal Hygiene and Veterinary Public Health, Leipzig University, Leipzig, Germany

**Keywords:** CPV-2, VP2 protein, Antigenic evolution, Viral fitness, Neutralization

## Abstract

Canine parvovirus type 2 (CPV-2) is a highly contagious pathogen responsible for severe gastroenteritis in carnivores, particularly in dogs. Continuous antigenic evolution has resulted in the emergence of CPV-2a, CPV-2b, and CPV-2c variants, defined by amino acid substitutions in the VP2 capsid protein. In this study, genetically altered CPV-2 viruses carrying common VP2 mutations (S297A, V300G, D305Y, Y324I, N426D, N426E, and T440A) were generated to evaluate their effects on viral behavior in vitro. Mutations were individually introduced into an infectious backbone (strain 447), classified as CPV-2a based on VP2 sequence analysis, and compared with the ancestral CPV-2 backbone (strain 265). Viral dynamics were assessed using quantitative PCR and immunofluorescence assays to examine extracellular viral DNA and intracellular infection, enabling discrimination between replication and virion release. Distinct profiles were observed among mutants, particularly for N426D, N426E, and T440A, and the impact of each substitution was influenced by the genetic background. Polyclonal antibodies induced by commercial CPV-2 vaccines neutralized both the CPV-2 control and the CPV-2 N426E mutant, indicating cross-reactivity. These findings demonstrate that VP2 evolution must be interpreted through functional approaches rather than solely by variant classification and provide experimentally based information relevant for molecular surveillance and vaccination strategies.

**Supplementary Information:**

The online version contains supplementary material available at 10.1007/s42770-026-02035-2.

## Introduction

Canine parvovirus type 2 (CPV-2) is a small, non-enveloped, single-stranded DNA virus belonging to the family *Parvoviridae*, genus *Protoparvovirus*, and species *Carnivore protoparvovirus*[1]. Since its emergence in the late 1970 s, CPV-2 has become one of the most important viral pathogens affecting domestic dogs, causing severe gastroenteritis and high mortality, particularly in young animals [2]. Remarkably, despite being a DNA virus, CPV-2 exhibits genomic substitution rates comparable to those of RNA viruses, reflecting its strong capacity for adaptation under selective pressures [3].

The CPV-2 genome encodes two non-structural proteins (NS1 and NS2) and two capsid proteins (VP1 and VP2), with VP2 constituting approximately 90% of the viral capsid [4]. As the main structural and antigenic component of the virion, VP2 plays a central role in host range determination, receptor binding, antigenicity, and viral fitness [5]. Consequently, amino acid substitutions within VP2 have been frequently associated with changes in viral infectivity, immune recognition, and epidemiological success.

Historically, CPV-2 has been described through the emergence of antigenic variants CPV-2a, CPV-2b, and CPV-2c, which differ mainly at residue 426 of the VP2 protein [6, 7]. Although substitutions at this site have been widely used for variant classification, accumulating evidence indicates that viral adaptation is not driven by a single residue, but rather by a set of mutations across functionally relevant regions of VP2. Several positions, including residues 297, 300, 305, 324, and 440, are located near or within structural motifs involved in capsid stability, receptor interaction, or antigenic loops, and have been shown to evolve under positive selection [8–12].

Despite the growing number of VP2 mutations described worldwide [12], the functional consequences of individual amino acid substitutions remain incompletely understood. Most available studies rely on sequence-based or epidemiological analysis, whereas relatively few have experimentally assessed how single VP2 mutations affect viral fitness and interaction with the host cell in a controlled genetic background [6, 13–15]. This gap limits our ability to interpret the biological relevance of newly emerging variants and to predict how specific substitutions may influence viral behavior beyond antigenic classification.

Viral fitness is a multifaceted trait encompassing not only genome replication, but also virion assembly, intracellular trafficking, and release efficiency [16]. Mutations that enhance one step of the viral life cycle may impair others, resulting in complex replication phenotypes that cannot be inferred solely from viral load measurements or antigenic profiles. Therefore, experimental systems based on infectious clones provide a powerful approach to disentangle the effects of individual mutations on viral fitness and replication dynamics [6].

In this study, we generated a panel of CPV-2 variants carrying common VP2 amino acid substitutions (S297Y, V300G, D305Y, Y324I, N426D, N426E, and T440A) individually introduced into an isogenic viral backbone. The effects of these mutations on viral fitness were assessed using quantitative PCR and immunofluorescence-based assays to evaluate extracellular viral DNA and intracellular infection. In addition, the neutralization capacity of polyclonal antibodies raised against commercial CPV-2 vaccines was examined to explore potential cross-reactivity with a representative variant. Together, these analyses aim to clarify the contribution of individual VP2 mutations to CPV-2 fitness and evolutionary dynamics.

## Materials and methods

### Construction of genetically altered viruses

An infectious plasmid clone designated as strain 447 (CPV-2a) was derived from a CPV field isolate obtained in 1995 [8]. This field isolate was cloned into a plasmid (pBI) and has since been maintained as an infectious clone in the laboratory. For comparison, an infectious plasmid clone corresponding to the ancestral CPV-2 strain 265 (GenBank accession number M38245.1) was included. Both plasmid-derived viruses were sequenced prior to mutagenesis, and VP2 amino acid differences between the two backbones are summarized in Supplementary TableS1.

**Fig. 1:**
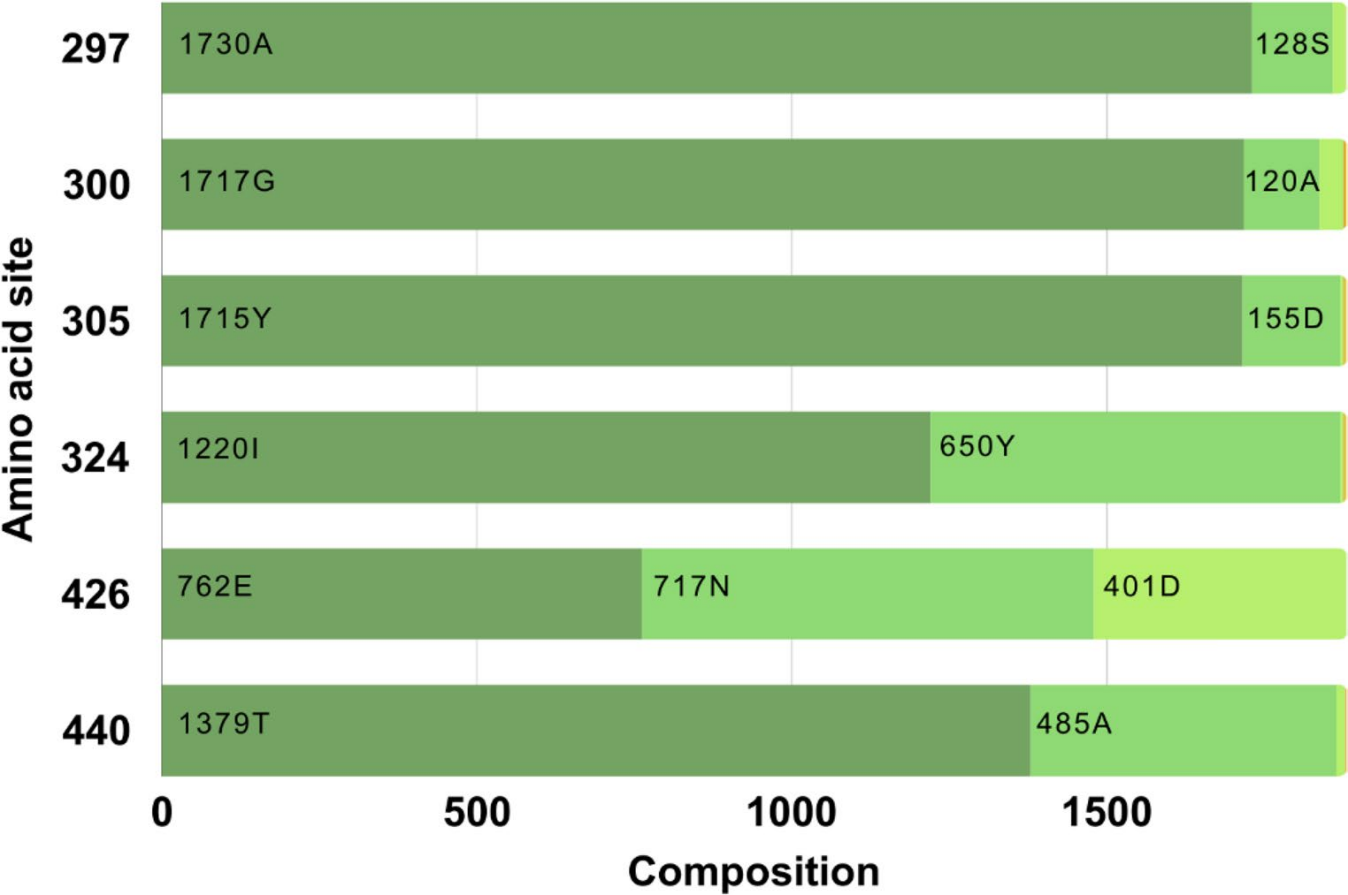
Distribution of amino acids at key VP2 protein sites from 2013 to 2024, based on 1.880 sequences available in GenBank. Only the most frequent variants are shown

Site-specific VP2 substitutions were individually introduced into the 447 backbone using a site-directed mutagenesis kit (Invitrogen, USA) with overlapping primers (Supplementary Table S2). The VP2 mutations S297A, V300G, D305Y, Y324I, N426D, N426E, and T440A were selected for evaluation due to their frequent occurrence in CPV-2 strains isolated worldwide from 2013 to 2024 (Fig. 1).

Competent *Escherichia coli* TOP10 cells (Invitrogen, USA) were transformed by heat shock, and double-stranded DNA concentrations were determined using a Qubit dsDNA BR Assay Kit and Qubit Fluorometer (Thermo Fisher Scientific, USA).

## Transfection and propagation

Feline kidney cells (CRFK) were seeded at a density of 1 × 10⁴ cells/mL 24 h prior in Dulbecco’s Modified Eagle’s Medium (DMEM) supplemented with 10% fetal bovine serum (FBS) penicillin (100 U/mL) and streptomycin (100 µg/mL). Transfection with the plasmids (each separately in triplicate) was performed usingLipofectamine™ 3000 reagent (Invitrogen, USA). A positive CPV-2 control (C+) and a negative control were included in all experiments. The positive control consisted of a field strain of CPV-2b, diluted 1:10 for validation of the experimental assays. The same positive control (C+) was used consistently in transfection, propagation, qPCR and virus neutralization assays.

For the negative control, nuclease-free water was used in place of the DNA template. After transfection, cells were incubated at 37 °C in a humidified atmosphere with 5% CO₂. To assess transfection efficiency, cells were fixed 48 h post-transfection with a 1:1 mixture of acetone and methanol, blocked with 3% FBS and incubated with polyclonal antibodies against CPV-2 (1:50). Positive cells were visualized using immunofluorescence with fluorescein-conjugated anti-canine VP2 antibody (1:100).

For viral propagation, CRFK cells were seeded 24 h prior at a density of 3 × 10³ cells/cm². The cells were washed once with phosphate-buffered saline (PBS), then 10 µL of each genetically altered virus, parental strains or positive CPV-2 control (C+) was added in triplicate and allowed to adsorb to the cells for 1 h at 37 °C. After adsorption, fresh DMEM was added and the cells were incubated further at 37 °C. Once the cells reached confluence, supernatants were collected. In the third passage, cells were seeded 24 h earlier (7 × 10⁴ cells/cm²) and 35 µL of each genetically altered virus, parental strains or positive CPV-2 control (C+) was added, this time in 12-well plates, following the same infection protocol. Once confluence was achieved, the entire volume from passage 3 was harvested and stored for subsequent experiments. DNA extraction from the supernatants was performed using the DNeasy Blood and Tissue Kit (Qiagen, USA).

At each passage, infected cells were visualized by immunofluorescence, while viral DNA from supernatants was extracted and quantified by real-time PCR (qPCR). This design allowed parallel assessment of intracellular infection and extracellular viral DNA. The qPCR assay targeting the VP2 gene was conducted using TaqMan-based detection, employing the following primer sequences: forward primer (5’-TGGAACTAGTGGCACACCAA-3’), reverse primer (5’-AAATGGTGGTAAGCCCAATG-3’) and probe (5’−6FAM-CAGGTGATGAATTTGCTACAGG-BHQ1-3’) [17]. Briefly, 3 µL of DNA, with concentrations determined by Qubit fluorometry (Supplementary Table S3), was added to a 17 µL reaction mixture containing TaqMan Master Mix, with final concentrations of 300 nM for the forward primer and 200 nM for the reverse primer and probe. The thermal cycling conditions included an initial denaturation at 95 °C for 3 min, followed by 40 cycles of denaturation at 95 °C for 10 s, and a combined annealing and extension step at 60 °C for 25 s. The same positive and negative controls, as previously described, were included in the assay. Additional informations regarding the first through third passages are available in the supplementary material (Fig.S1).

Stock viruses were titrated using an immunofluorescence-based 50% tissue culture infective dose (TCID₅₀) assay in CRFK cells [6].

## Infectivity assay

CRFK cells were seeded at a density of 1 × 10³ cells/cm² in 96-well plates and infected at a multiplicity of infection (MOI) of 0.1 TCID₅₀ [6], in triplicate. Supernatants were harvested at 48- and 72-hours post-infection (hpi) and plates were stained using the immunofluorescence assay described above. Infected cells in all wells were counted. Additionally, genetic material from the supernatants was extracted and qPCR was performed as mentioned previously. The results of the genetically altered viruses were compared with those of the parental strains 265 and 447 and the previously described positive CPV-2 control (C+).

## Generation of polyclonal antibodies

Thirty rats were immunized with six commercial vaccines to generate polyclonal antibodies against CPV-2. The rats were divided into groups of five, with an additional negative control group. Each group received three booster injections at 15-day intervals, while the negative control group received saline. Blood samples were taken before each booster, and the final collection was performed 15 days after the last booster. Serum samples were heat-inactivated at 56 °C for 30 min and stored at −20 °C. This study was approved by the Ethical Committee on the Use of Animals of UCS (protocol number 06/2022).

## Virus neutralization assay

All serum samples from the rats were serially diluted in 5-log steps, with 0.1 mL of each dilution mixed with an equal volume of 200 TCID₅₀, following the neutralization protocol described by Streck et al. [14], using either the positive CPV-2 control (C+) or the genetically altered virus CPV-2 N426E. After 2 h of incubation at 37 °C, 100 µL of the serum-virus mixture was added to CRFK cells seeded 24 h prior at a density of 1 × 10⁴ cells/mL in 96-well plates, in triplicate. The cells were grown in DMEM supplemented with penicillin and streptomycin and the plates were incubated for 5 days at 37 °C in a humid environment containing 5 % CO₂. After the incubation period, the cells were fixed and the immunofluorescence assay was performed as described earlier.

### Statistical analyses

The data were analyzed using SPSS software (IBM, USA). For comparison of infected cell counts among groups, a one-way ANOVA was performed, and when significant differences were detected, Tukey’s post hoc test was applied for pairwise comparisons, adopting a significance level of *p* < 0.05. For virus neutralization (VN) analyses, the paired Wilcoxon test was used with the same significance threshold (*p* < 0.05).

## Results and discussion

Canine parvovirus type 2 (CPV-2) has circulated globally for more than four decades and continues to diversify under complex selective pressures [4]. In the present study, all genetically altered constructs and the two parental backbones were successfully transfected into CRFK cells, resulting in the recovery of infectious virus. The maintenance of viral DNA through three serial passages, quantified by fluorometry (Fig.S1 and Table S3), confirmed that all mutants remained viable and suitable for comparative evaluation.

Viral behavior was assessed using two complementary approaches. Quantitative PCR was performed on culture supernatants, and therefore Ct values represent extracellular viral DNA, providing an indirect estimate of viral release. Immunofluorescence, in contrast, detects viral protein expression within cells and reflects intracellular infection. This strategy enabled the simultaneous evaluation of infection and release, offering a more comprehensive view of viral dynamics than either method alone.

**Fig. 2:**
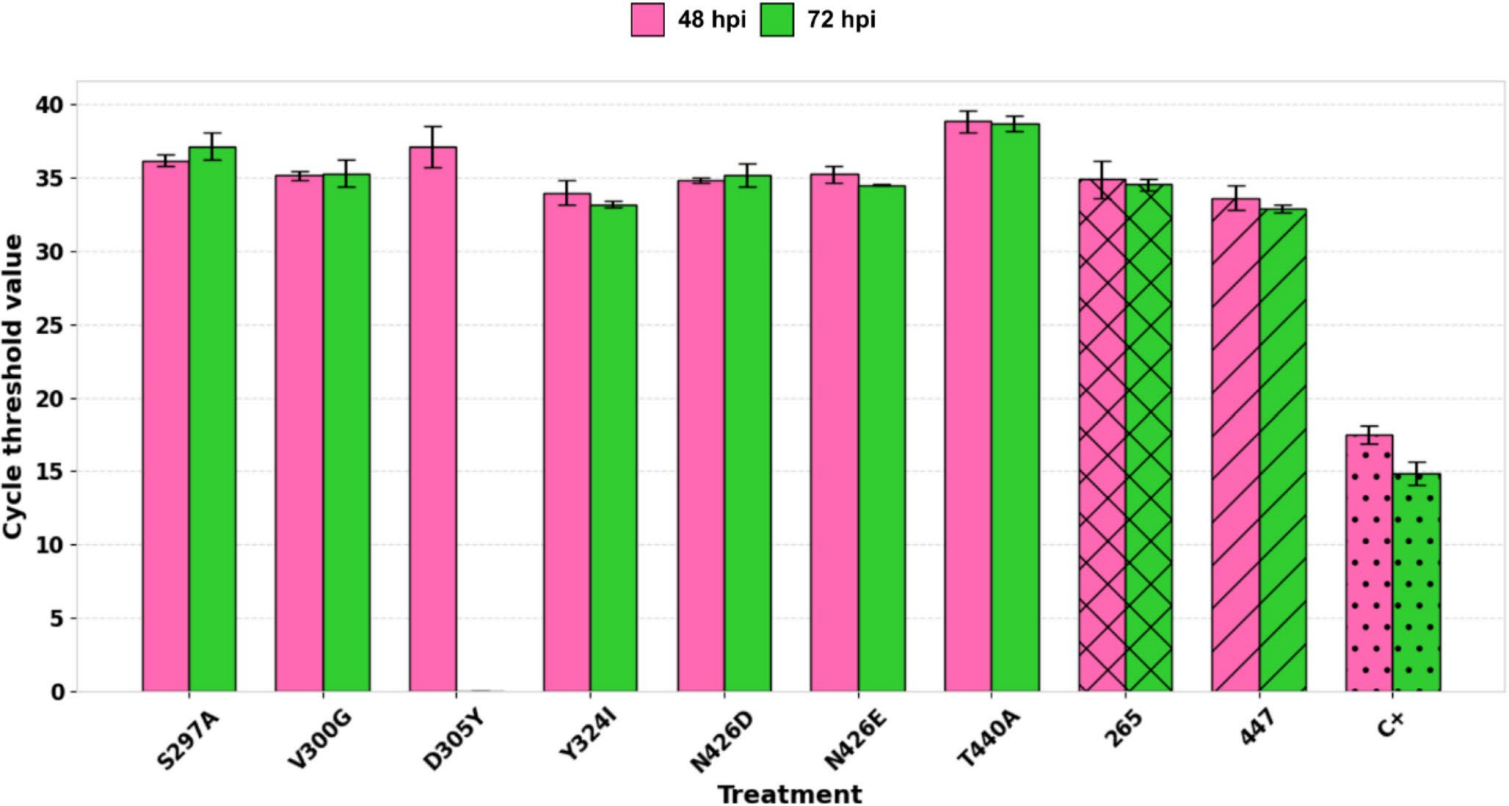
Replication of the CPV-2 genetically altered viruses, parental strains and positive CPV-2 control (C+) as cycle threshold (Ct) values measured by qPCR at 48 and 72 hpi from the supernatant. Lines represent the mean ± standard deviation

**Fig. 3:**
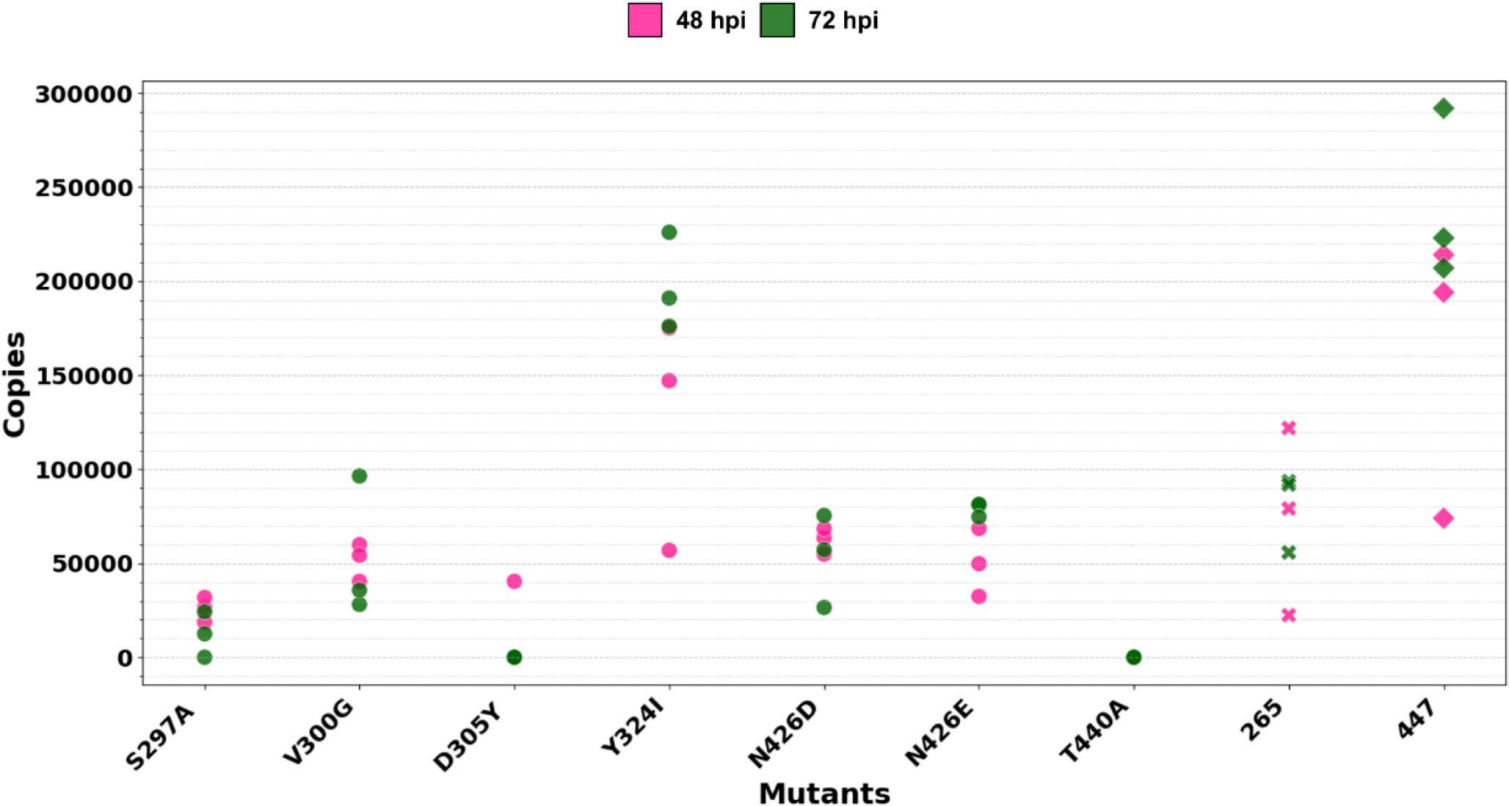
Replication of the CPV-2 genetically altered viruses and parental strains expressed as viral genome copy equivalents measured by qPCR at 48 and 72 hpi from the supernatant

**Fig. 4:**
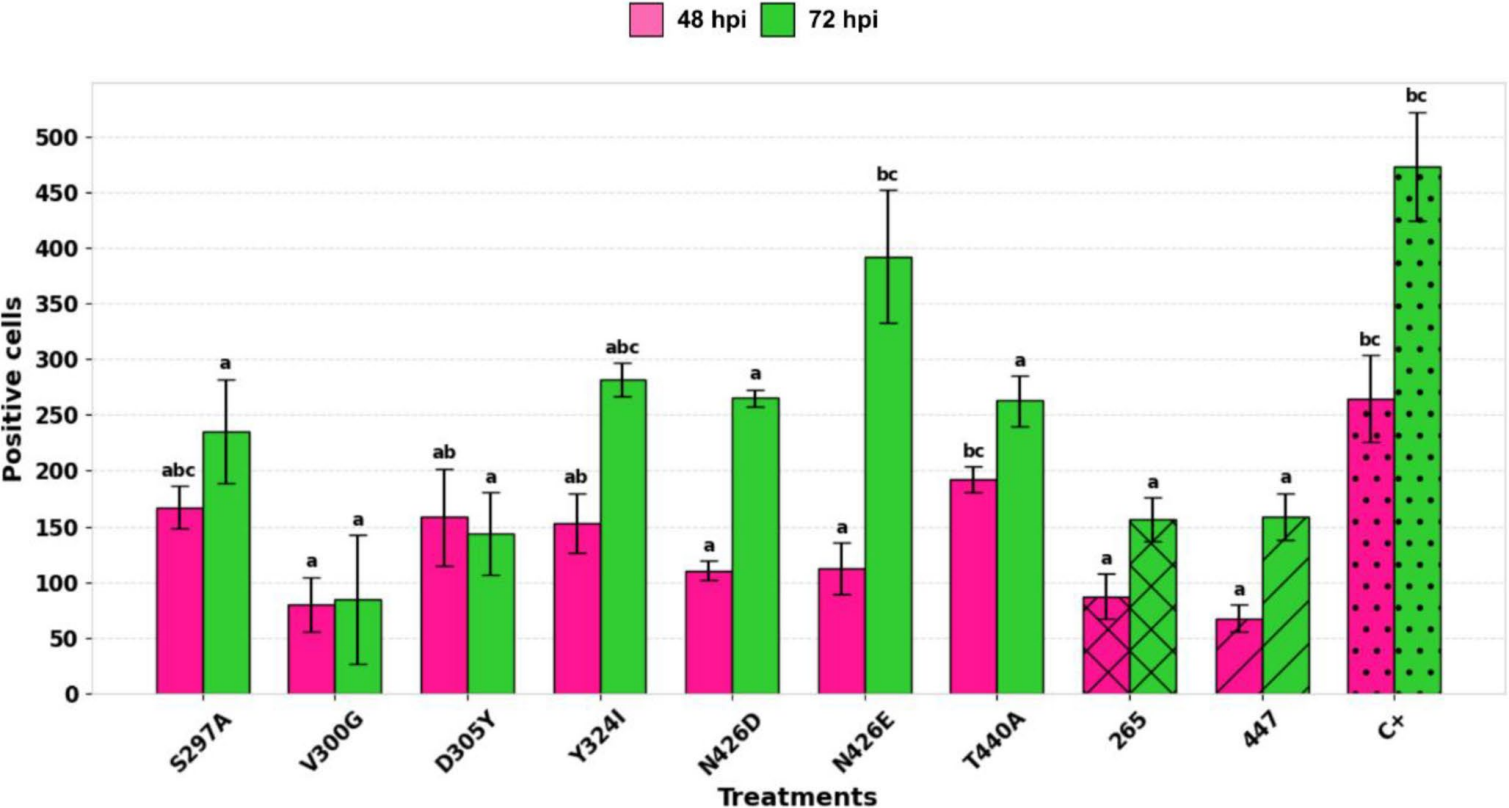
Replication of CPV-2 genetically altered viruses, parental strains and the positive CPV-2 control (C+) represented by the number of positive cells obtained through immunofluorescence at 48 and 72 hpi. Lines expressed the mean ± standard deviation. Means were compared using a unidirectional ANOVA followed by Tukey’s post hoc test (p < 0.05). The small lowercase letters (‘a’, ‘b’, ‘c’) annotated on top of the bars indicate statistically significant differences (p < 0.05) from specific positive controls, as determined by Tukey’s post hoc test. These annotations apply to the specific time point (48 hpi or 72 hpi) of the bar they are placed upon, indicating a significant difference for that time point. a: Significant difference compared to C+. b: Significant difference compared to 447. c: Significant difference compared to 265

qPCR analyses at 48 and 72 hpi are summarized in Fig. 2 as mean Ct values, whereas Fig. 3 presents the same data expressed as genome copy numbers with individual triplicates, highlighting the variability observed among replicates. Several constructs displayed high Ct values or low copy numbers in the supernatant while still presenting numerous fluorescent-positive cells (Fig. 4), indicating that some mutants may remain predominantly cell-associated, with limited efficiency of virion maturation or egress rather than being replication-defective. The presentation of individual replicates in Fig. 3was included to illustrate the inconsistent extracellular DNA profiles that contrasted with the more stable immunofluorescence results. Statistical comparisons of infected cell counts obtained by immunofluorescence are shown in Fig. 4. Such dissociations between intracellular infection and extracellular viral load have been conceptually discussed in the context of viral fitness measurements [16].

Residues 297, 300, 305 and 324 are located near the three-fold axis of the capsid, a region implicated in receptor interaction and host specificity [8, 9]. The substitutions S297A and Y324I, which are widely distributed among contemporary field strains [18, 19], showed replication patterns comparable to or slightly higher than those of the parental backbones at specific time points. These observations agree with previous reports indicating that modifications within this region can modulate viral entry and cell tropism [6]. The broader concept that transitional or intermediate mutations may facilitate host adaptation has been experimentally demonstrated [13], supporting the rationale for evaluating individual VP2 substitutions within defined infectious clones rather than relying solely on variant labels.

Residues 426 and 440 are positioned within loop 4 of VP2, one of the most immunogenic domains of the capsid [20]. Although residue 426 has historically been used for classification of CPV-2a, 2b and 2c [12], accumulating evidence indicates that viral phenotype results from the combined effect of multiple sites. In our assays, mutants N426D, N426E and T440A displayed distinct behavior at 72 hpi relative to both parental backbones. The non-conservative nature of these substitutions may influence capsid stability or antigenic structure [12], as previously suggested for T440A in the context of antigenic drift [20]. Importantly, the impact of each mutation differed according to the genetic background, highlighting that the phenotypic expression of a substitution cannot be fully predicted without considering the surrounding sequence [14].

Neutralization assays were performed to determine whether the N426E mutation could affect recognition by vaccine-induced antibodies. Sera from rats immunized with six commercial vaccines neutralized both the CPV-2 control and the CPV-2 N426E mutant with titers ≥ 1:80 (equivalent to ≥ 6.32 log₂) [21] (Table1). Comparison of neutralization titers using the paired Wilcoxon test showed no statistically significant differences between the control and the N426E mutant for any vaccine (*p*> 0.05). Although minor variations in mean Log₂ titers were observed, the overall antibody reactivity against the mutant was comparable to that against the control virus, supporting cross-reactivity rather than antigenic escape [22, 23]. It should be considered that live CPV-2 vaccines are not expected to replicate efficiently in rats, therefore antibody levels may underestimate responses in the natural canine host. Divergent results among previous studies [24, 25] likely reflect differences in vaccine composition, animal models and assay conditions.

**Table 1:** Neutralization activity of polyclonal serum samples from rats immunized with six different vaccines against 200 TCID₅₀ of the positive CPV-2 control (C+) and the genetically altered virus CPV-2 N426E. Results are expressed as Log₂ mean ± SD. Statistical comparison between C + and N426E was performed using the paired Wilcoxon test; no significant differences were detected (*p* > 0.05)

Vaccine	Positive CPV-2 control Log_2_ mean ± SD	CPV-2 N426E Log_2_ mean ± SD
1	8.92 ± 0.5	9.52 ± 0.4
2	8.92 ± 1.1	8.32 ± 1
3	8.52 ± 1.6	8.32 ± 0.7
4	9.92 ± 0.5	9.12 ± 0.4
5	7.57 ± 1.0	7.32 ± 0.8
6	8.12 ± 1.0	7.92 ± 1.1

The use of two infectious backbones with distinct VP2 profiles represented a central aspect of this work. Strain 447, originally derived from a CPV-2b field isolate and currently classified as CPV-2a based on updated sequencing, and the ancestral strain 265 provided complementary genetic contexts for evaluating mutational effects. The observed differences between these backbones reinforce the concept that VP2 evolution operates through complex interactions among residues, as proposed in structural and evolutionary analyses [12, 13].

This study has limitations that should be acknowledged. All experiments were conducted in vitro, and viral behavior in cell culture may not fully reproduce dynamics in naturally infected dogs. qPCR analyses were based on supernatants and therefore reflect extracellular DNA rather than total replication. In addition, neutralization was assessed only for the N426E mutant, limiting conclusions regarding other substitutions. Future studies addressing combinations of mutations, receptor-binding affinity and in vivo models will be essential to further elucidate these mechanisms.

Taken together the results indicate that the effects of VP2 substitutions manifest at multiple levels of the viral cycle and may not be directly reflected by a single readout. The divergence between qPCR and immunofluorescence underscores the importance of distinguishing intracellular infection from extracellular viral DNA when interpreting CPV-2 replication in vitro.Likewise, the distinct behavior of the same mutation in backbones 265 and 447 reinforces that phenotypic outcomes are context-dependent. These aspects should be considered in future functional studies and in the interpretation of sequence-based surveillance data.

## Conclusions

Individual substitutions in VP2 modulated the behavior of CPV-2 in vitro in a measurable and context-dependent manner, indicating that the viral phenotype cannot be inferred solely by the traditional designation of variants. The combined application of qPCR and immunofluorescence proved essential to distinguish intracellular infection from extracellular viral DNA, revealing that mutations such as N426D, N426E, and T440A may predominantly affect virion release rather than replication itself. Comparison of two infectious structures demonstrated that the outcome of a given substitution is strongly influenced by the surrounding genetic context. Furthermore, vaccine-induced antibodies maintained neutralizing activity against the N426E mutant, corroborating the antigenic relationship between circulating variants. These results highlight the value of functional approaches for interpreting VP2 evolution and provide experimentally based information relevant to CPV-2 surveillance and the ongoing evaluation of vaccination strategies.

## Supplementary Information

Below is the link to the electronic supplementary material.

Supplementary Material 1 (DOCX 588 KB) 

## Data Availability

Data available on request from the authors.
