# Supplementary material for: Impact of VP2 mutations on viral fitness in canine parvovirus
Source: Braz J Microbiol. 2026 Jul 27;57(1):219. doi: 10.1007/s42770-026-02035-2 (PMC13407408; doi:10.1007/s42770-026-02035-2)
Supplement: Supplementary file 1 — Supplementary Material 1 (DOCX 588 KB) [file 42770_2026_2035_MOESM1_ESM.docx]

**Supplementary material**

**Supplementary table S1.** Amino acid differences in the VP2 protein between parental strains 265 and 447

| **Parental strains** | **Amino acid profile in VP2** | | | | | | | | | |
| --- | --- | --- | --- | --- | --- | --- | --- | --- | --- | --- |
|  | **93** | **101** | **217** | **232** | **300** | **323** | **375** | **411** | **564** | **568** |
| 265 | N | I | T | I | A | N | N | E | S | G |
| 447 | K | T | A | V | V | D | D | A | N | A |

#### **Supplementary table S2.** Sequences of the primers used for site-directed mutagenesis and their respective amino acid target in the VP2 protein

| **Amino acid target** | **Sequence (5′ to 3′)** |
| --- | --- |
| 297 | CTAAATTCTTTGCCTCAATCTGAAGGAGGTACTAAC |
| 300 | GCCTCAATCTGAAGGAGGTACTAACTTTGGTGATA |
| 305 | GGAGTTACTAACTTTGGTTATATAGGAGTTCAAC |
| 324 | CAAATGGGAAATACAAACATTATTACTGAAGCTACTATT |
| 426D | CTTTAACCTTCCTGTAACAAATGATAATGTATTGCTACC |
| 426E | CTTTAACCTTCCTGTAACAGAAGATAATGTATTGCTACC |
| 440 | GATCCAATTGGAGGTAAAGCAGGAATTAACTATACTAAT |


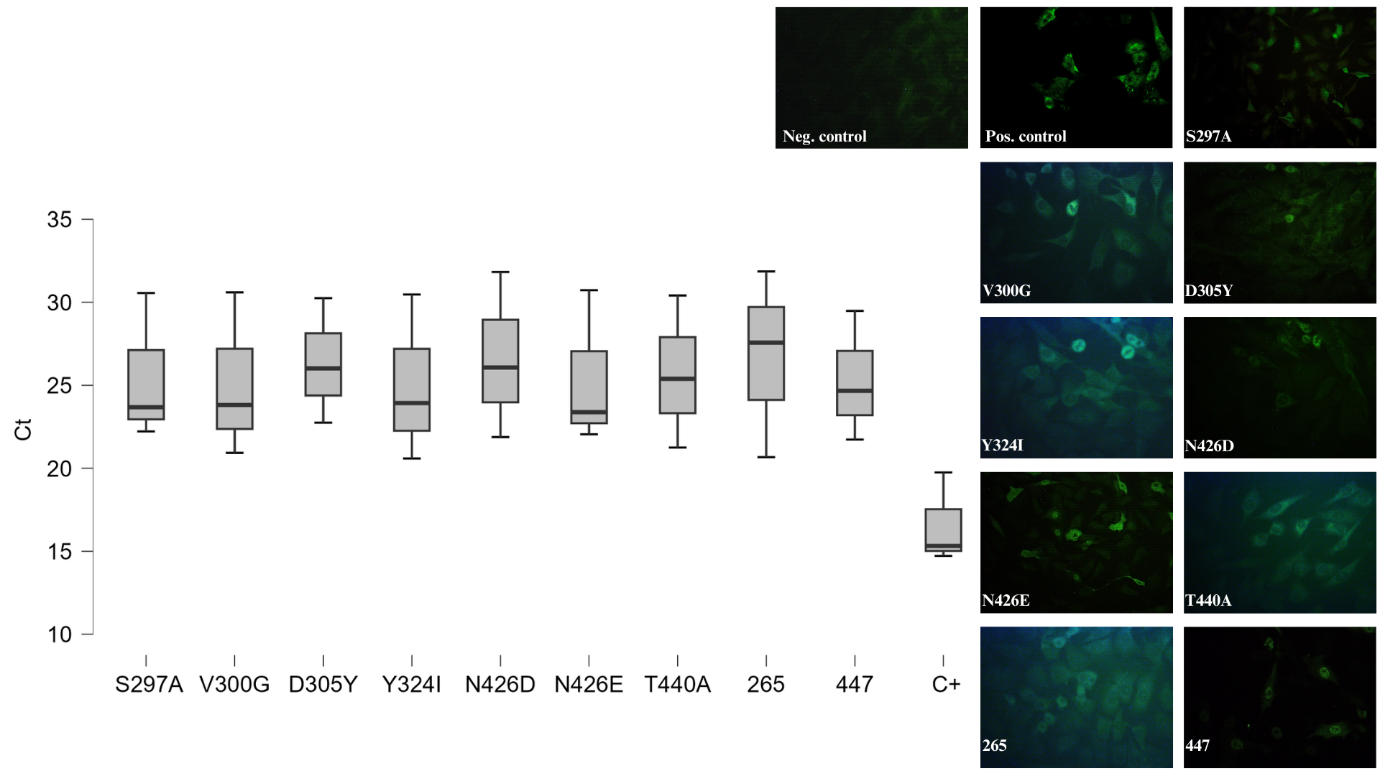


**Supplementary figure S1.** Graph showing the cycle threshold (Ct) values of the mutants, parental strains and positive CPV-2 control (C+) over the first to third passages. In the adjacent images, the results of immunofluorescence from the third passage of mutants, parental strains, positive CPV-2 control and negative control are presented.

**Supplementary table S3.** Viral DNA quantification after the third cell culture passage of VP2 mutant viruses, parental strains, and the CPV-2 positive control (C+), as measured by Qubit fluorometric assay.

| **Sample** | **DNA (ng/µL)** |
| --- | --- |
| S297A | 72.00 |
| V300G | 73.70 |
| D305Y | 83.40 |
| Y324I | 71.70 |
| N426D | 74.10 |
| N426E | 79.10 |
| T440A | 158.00 |
| 265 | 150.00 |
| 447 | 67.80 |
| Positive CPV-2 control (C+) | 206.00 |
